# Rapidly Progressive Necrotizing Fasciitis of the Lower Limb Following Self-Drainage of an Abscess: Prompt Recognition and Initial Management at a District Healthcare Centre

**DOI:** 10.7759/cureus.91309

**Published:** 2025-08-30

**Authors:** Mark Salib, John Salib, Elisabeth A Hildenbrandt, Mark E Hoffman, Frederick Tiesenga

**Affiliations:** 1 School of Medicine, St. George's University School of Medicine, St. George's, GRD; 2 Department of General Surgery, West Suburban Medical Center, Chicago, USA

**Keywords:** case report, group a streptococcus, necrotizing fasciitis (nf), necrotizing soft tissue infection (nsti), surgical debridement

## Abstract

Necrotizing fasciitis (NF) is a rare but rapidly progressive infection of the fascia and subcutaneous tissue that can arise following trauma, surgical wounds, or minor skin breaches. Early stages often mimic cellulitis, making timely diagnosis challenging. This case report describes a 52-year-old male patient who presented with erythema and swelling of the left foot and ankle, initially diagnosed as cellulitis in the setting of septic shock. Despite nonspecific imaging and laboratory findings, the rapid progression of cutaneous changes raised clinical suspicion for NF. Emergent surgical debridement confirmed the diagnosis, and the patient was managed postoperatively in the intensive care unit with broad-spectrum antibiotics and serial wound care. This case highlights the limitations of early diagnostic tools, emphasizes the importance of maintaining a high index of suspicion, and demonstrates how timely surgical intervention can significantly improve patient outcomes with NF.

## Introduction

Necrotizing fasciitis (NF) is a rare but highly aggressive soft tissue infection characterized by rapid necrosis of the fascia and subcutaneous tissue. It often presents as a polymicrobial infection, although monomicrobial cases involving *Streptococcus pyogenes* (Group A *Streptococcus*) or *Clostridium *species are also well documented [1, 2]. NF carries a significant risk of morbidity and mortality, with mortality rates ranging from 20% to over 40%, depending on the time of diagnosis and initiation of treatment [2].

Giuliano et al. classified NF according to the extent of tissue involvement, ranging from disease confined to the skin and subcutaneous tissue (Type I) to progressive spread into fascia (Type II), muscle (Type III), and visceral structures (Type IV) [3]. Wong et al. later introduced a microbiological classification, distinguishing polymicrobial infections involving mixed aerobic and anaerobic organisms (Type I) from monomicrobial infections, most commonly caused by Group A *Streptococcus *with or without *Staphylococcus aureus* (Type II) [4]. Together, these frameworks emphasize both the anatomical severity and the etiological spectrum of NF, providing complementary guidance for surgical management and empiric antimicrobial therapy.

Epidemiologically, NF is estimated to occur in 0.24 to 0.4 cases per 100,000 individuals annually. However, these numbers may underestimate the true incidence due to underreporting and frequent misdiagnosis, particularly in the early stages [1]. Patients with a greater risk burden include those with advanced age, diabetes mellitus, chronic kidney disease, malignancy, immunosuppression, or recent trauma, although healthy individuals may also be affected [3]. The Laboratory Risk Indicator for Necrotizing Fasciitis (LRINEC) score is a biochemical tool that uses routine laboratory parameters, such as C-reactive protein, white blood cell count, hemoglobin, sodium, creatinine, and glucose, to help differentiate NF from other soft tissue infections. More recently, the SIARI score, short for Site, Age, Immunosuppression, Renal impairment, and Inflammatory markers, has been proposed as a simpler clinical tool with improved predictive value, particularly for early identification in resource-limited settings [3-5].

The early clinical manifestations of NF are often nonspecific, resembling more common and benign conditions such as cellulitis or simple abscesses. This diagnostic ambiguity can lead to potentially hazardous delays in treatment. Classic findings, such as erythema, swelling, severe pain disproportionate to physical findings, bullae, crepitus, or skin discoloration, may not be present initially, further complicating early recognition [3, 4]. As the infection progresses, systemic symptoms such as hypotension, fever, and altered mental status can develop rapidly, underscoring the importance of a high index of suspicion in at-risk patients.

The cornerstone of NF management is early and aggressive surgical debridement, accompanied by broad-spectrum intravenous antibiotics and supportive care. Imaging modalities such as computed tomography (CT) scans or magnetic resonance imaging (MRI) may assist in evaluating the extent of soft tissue involvement. However, they should not delay operative intervention when clinical suspicion is high [4, 5]. This report describes a case of lower extremity NF initially presenting as cellulitis, highlighting the diagnostic and therapeutic challenges associated with this potentially fatal condition.

## Case presentation

A 52-year-old male patient presented to the emergency department with erythema and swelling of the left ankle and foot after attempting to drain a suspected abscess at home using a needle. The patient attempted self-drainage of a lower limb abscess using a safety pin that had been superficially heated with a lighter, without appropriate disinfection of the surrounding skin or instrument. Following the puncture, purulent material was manually expressed, and the wound was left open to facilitate presumed ongoing drainage. He reported difficulty bearing weight on the affected limb but denied systemic symptoms such as fever, chills, shortness of breath, chest pain, palpitations, abdominal pain, nausea, or vomiting. His past medical history included psoriasis, a prior gunshot wound, and alcohol use disorder. He had no known allergies.

**Figure 1:**
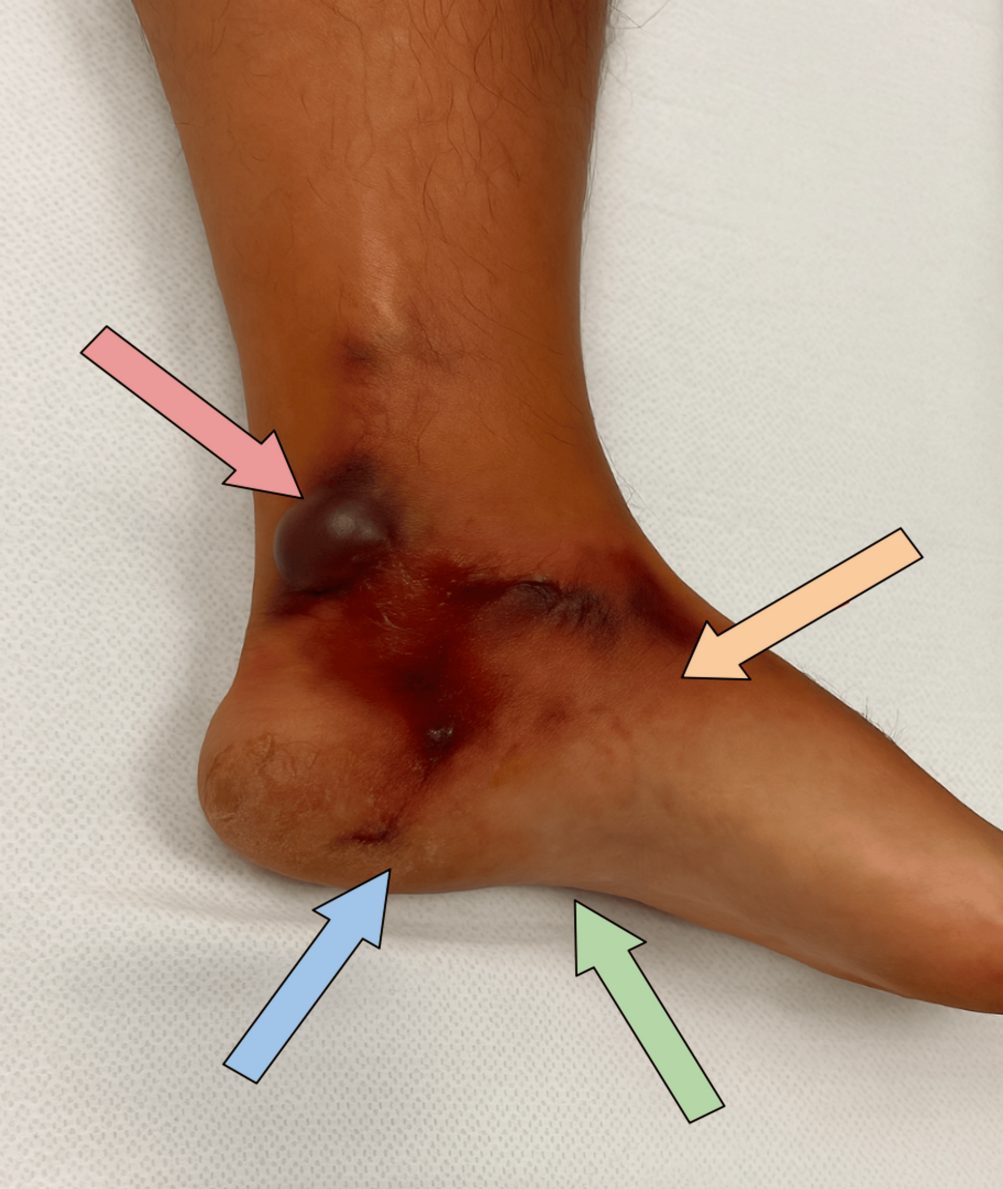
Clinical photograph of the left ankle and foot Clinical photograph of the left ankle and foot demonstrating hallmark findings of necrotizing fasciitis. Notable are areas of ecchymosis (blue arrow), skin discoloration (orange arrow), and a tense hemorrhagic bulla overlying the lateral ankle (red arrow). Surrounding soft tissue edema and early necrosis are evident (green arrow), indicating rapid progression of deep soft tissue infection. These features correlate with underlying soft tissue gas seen on imaging and are concerning for a limb-threatening infection.

On arrival, the patient appeared fatigued and in mild distress. Following the administration of morphine, his systolic blood pressure decreased to 70 mmHg. Intravenous fluids and empiric antibiotics were administered, and vasopressor support was initiated to maintain hemodynamic stability. Physical examination revealed poorly demarcated erythema and tenderness along the medial aspect of the left foot and ankle, with generalized inflammation extending toward the knee (Figure 1). Blistering and scabbing were noted around the ankle. Compartments were soft, and distal pulses were intact and symmetrical.

**Figure 2:**
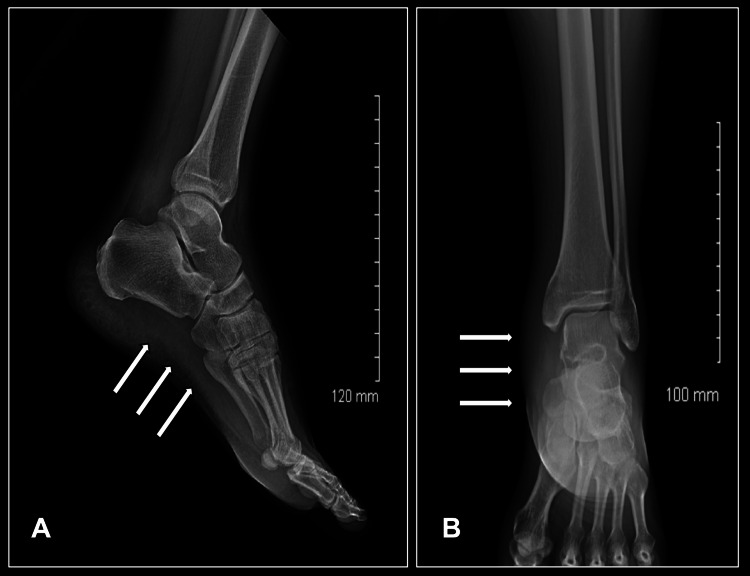
Plain radiographs of the left ankle and foot (A) Lateral view showing soft tissue swelling (white arrows) over the posterior and plantar aspects of the ankle; (B) Anteroposterior view demonstrating soft tissue gas (white arrows) tracking along the lateral subcutaneous and fascial planes, consistent with necrotizing fasciitis. No osseous abnormalities are noted.

**Figure 3:**
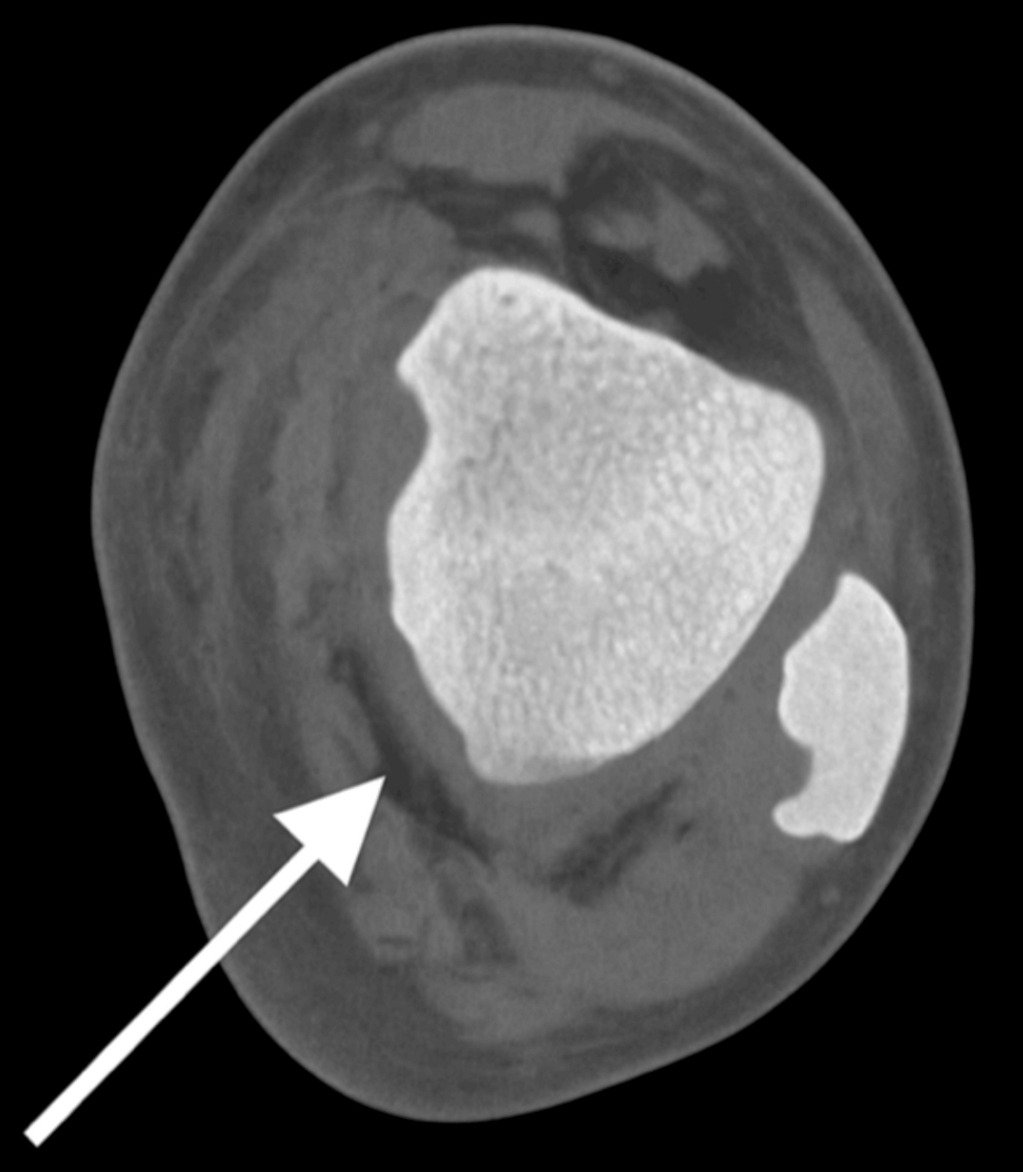
Axial CT scan of the left distal calf demonstrating early soft tissue changes in a case of necrotizing fasciitis A non-contrast axial computed tomography (CT) scan at the level of the left distal calf showing soft tissue stranding and haziness along the deep posterior compartment (white arrow), consistent with early fascial inflammation. No subcutaneous gas is visualized in this slice. These findings, though subtle, highlight the importance of clinical correlation in suspected necrotizing fasciitis, where imaging may appear equivocal in early stages.

Laboratory studies (Table 1) showed leukocytosis (WBC 13.8 ×10³/μL; normal range 4.0-11.0), neutrophilia (12.8 ×10³/μL; normal range 1.7-7.7 ×10³/μL), and elevated serum lactate (3.7 mmol/L; normal range <2 mmol/L). Imaging, including ultrasound, X-ray (Figure 2), and CT of the lower extremity (Figure 3). The x-ray revealed soft tissue swelling and slight gas formation, and no signs of myositis or osteomyelitis. Blood and wound cultures were positive for Group A *Streptococcus*. 

**Table 1:** Summary of the patient's initial laboratory evaluation on admission This table displays the patient's laboratory values with corresponding reference ranges. CMP assesses metabolic, renal, and hepatic function; CBC evaluates red and white blood cell indices and platelets; WBC differential outlines leukocyte subtypes for diagnostic interpretation.

Section	Test Name	Result	Reference Range
Complete Metabolic Panel (CMP)	Total Protein	5.2	6.0-8.3 g/dL
	Glucose	141	70-99 mg/dL
	Blood Urea Nitrogen (BUN)	14	7-20 mg/dL
	Creatinine	1.2	0.6-1.3 mg/dL
	Sodium	139	135-145 mmol/L
	Potassium	4	3.5-5.1 mmol/L
	Chloride	106	98-107 mmol/L
	Carbon Dioxide (CO2)	26	22-29 mmol/L
	Anion Gap	7	8-16 mmol/L
	BUN/Creatinine Ratio	12	10-20
	Calcium	7.9	8.5-10.5 mg/dL
	Phosphorus	2.7	2.5-4.5 mg/dL
	Albumin	3.7	3.5-5.0 g/dL
	Aspartate Aminotransferase (AST)	53	10-40 U/L
	Alanine Aminotransferase (ALT)	49	7-56 U/L
	Alkaline Phosphatase	40	44-147 U/L
	Total Bilirubin	0.1	0.1-1.2 mg/dL
	Glomerular Filtration Rate (GFR)	>60	≥60 mL/min/1.73m²
	Magnesium	2	1.7-2.2 mg/dL
Complete Blood Count (CBC)	White Blood Cells (WBC)	13.8	4.0-11.0 x10^3/µL
	Red Blood Cells (RBC)	3.45	4.2-5.4 x10^6/µL
	Hemoglobin (Hgb)	11	12-16 g/dL
	Hematocrit (Hct)	32.5	36-46%
	Mean Corpuscular Volume (MCV)	94.1	80-100 fL
	Mean Corpuscular Hemoglobin (MCH)	32	27-33 pg
	Mean Corpuscular Hemoglobin Concentration (MCHC)	34	32-36 g/dL
	Red Cell Distribution Width (RDW)	13.9	11.5-14.5%
	Platelets	247	150-450 x10^3/µL
	Mean Platelet Volume (MPV)	8.2	7.5-11.5 fL
CBC Differential	Lymphocytes %	10	20-40%
	Monocytes %	4	2-8%
	Eosinophils %	7	1-4%
	Segmented Neutrophils (Segs) Absolute	12.8	1.7-7.7 x10^3/µL
	Bands Absolute	1.1	0-0.7 x10^3/µL
	Lymphocytes Absolute	1.1	1.0-4.8 x10^3/µL
	Monocytes Absolute	0.5	0.2-0.8 x10^3/µL
	Eosinophils Absolute	0.8	0.0-0.5 x10^3/µL

**Figure 4:**
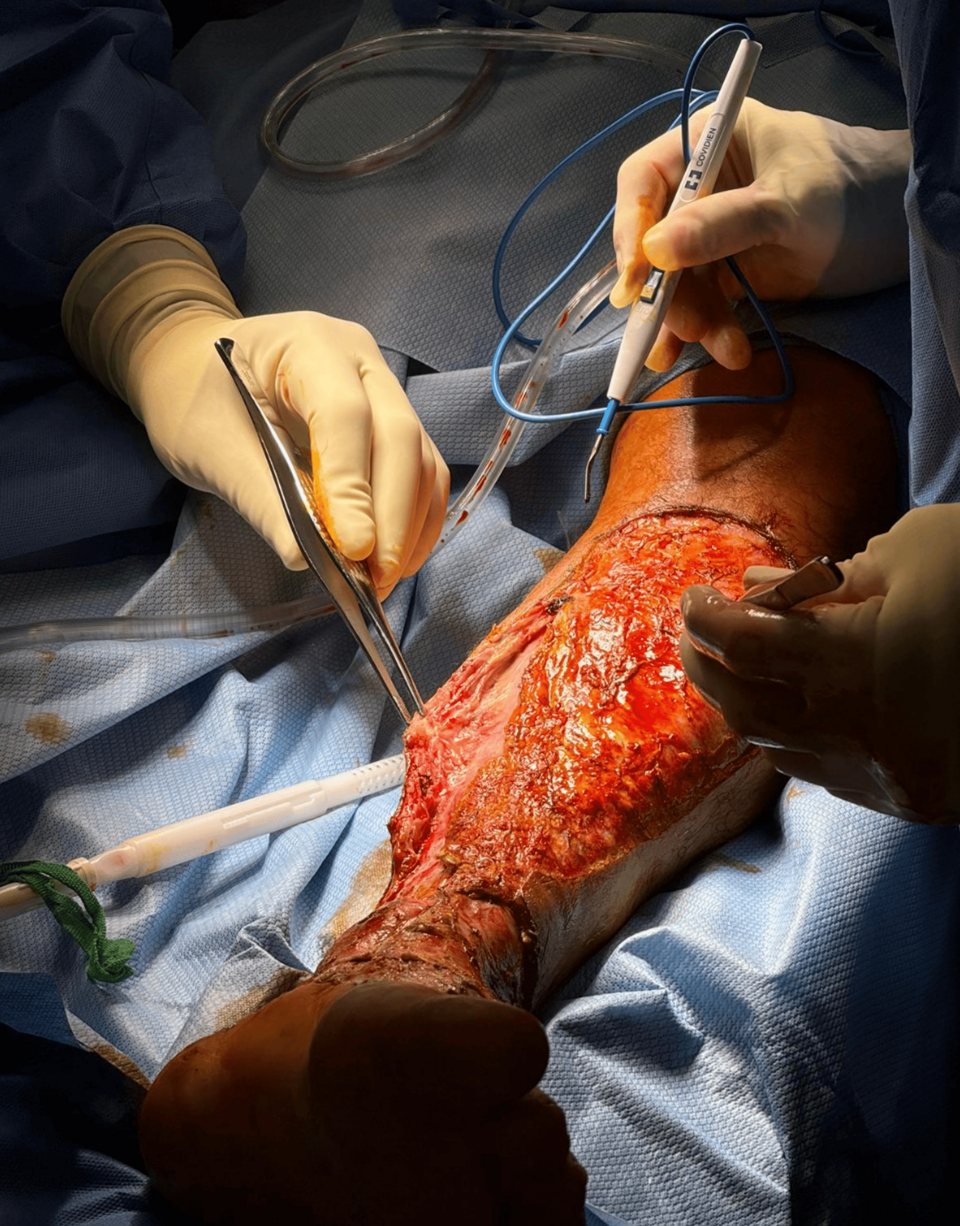
Surgical debridement of the left lower extremity in the patient diagnosed with necrotizing fasciitis Intraoperative photograph showing extensive surgical debridement of the left lower extremity in a patient with necrotizing fasciitis. Non-viable skin, subcutaneous tissue, and fascia have been excised, revealing underlying healthy muscle. Early aggressive debridement is critical in halting the progression of necrotizing soft tissue infections and improving outcomes.

An initial diagnosis of cellulitis was made. However, due to the rapid progression of skin changes and clinical deterioration, a surgical consultation was obtained. Broad-spectrum empiric antimicrobial therapy was initiated with linezolid, piperacillin-tazobactam, cefepime, and clindamycin to ensure coverage against methicillin-resistant *Staphylococcus aureus* (MRSA), Gram-negative bacilli, anaerobes, and toxin-producing *Streptococcus pyogenes*. Clindamycin was specifically included for its role in suppressing streptococcal toxin and cytokine production. Antibiotics were continued for approximately 14 days. The patient underwent urgent surgical debridement of the left lower limb involving the skin, fascia, and muscle (Figure 4). Postoperatively, he was admitted to the intensive care unit and managed under a sepsis protocol. The wound was packed and closely monitored. Multiple subsequent debridements were performed over the following days to control the infection and promote healing. Once the patient was stabilized and initial surgical and antimicrobial measures were undertaken, the patient was transferred to a tertiary care facility for ongoing multidisciplinary management. His course included repeat debridements, definitive wound closure with split-thickness skin grafting, and extensive physical and occupational therapy aimed at restoring mobility, function, and independence.

**Figure 5:**
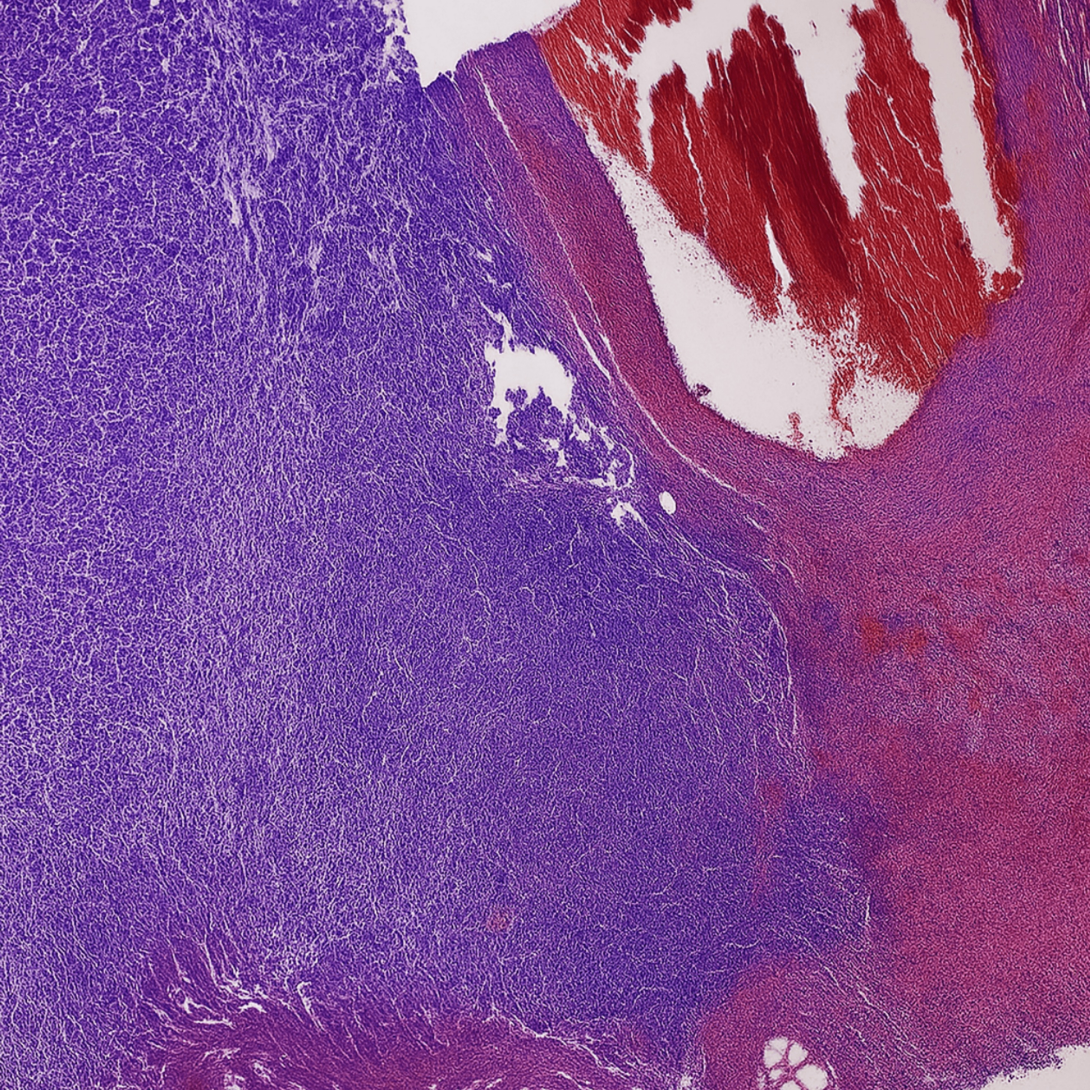
Hematoxylin and eosin (H&E) stained section of soft tissue demonstrating features of necrotising fasciitis (20X magnification). Histopathological examination reveals fibro-adipose tissue with extensive areas of necrosis, dense acute and chronic inflammatory cell infiltrates, abscess formation, and hemorrhagic changes. Reactive tissue changes are present, but no evidence of malignancy is seen. These findings are consistent with necrotizing fasciitis.

A tissue sample from the patient’s left lower leg was submitted to the pathology laboratory for analysis. Gross examination revealed three tan-whitish soft tissue fragments measuring 2.4 × 1.8 × 0.2 cm. As seen in this case, the histopathologic evaluation (Figure 5) demonstrated fibro-adipose and vascular tissue with extensive necrosis, acute and chronic inflammatory infiltrates, abscess formation, and hemorrhagic changes. Reactive changes were also noted. These findings, further illustrated in Figure 5, are consistent with a significant infectious or inflammatory process. No evidence of malignancy was identified in the examined tissue.

## Discussion

NF is a severe, rapidly progressing infection of the soft tissues that primarily involves the fascia and subcutaneous layers. It predominantly affects individuals with predisposing factors such as advanced age, peripheral vascular disease, diabetes mellitus, obesity, or immunocompromised states [4-6]. Although trauma, surgical wounds, or penetrating injuries are common antecedents, NF can also develop following minor skin injuries such as scratches, insect bites, or abrasions. This variability in presentation underscores the importance of maintaining a high index of suspicion even in patients without obvious risk factors [7, 8].

NF is a rapidly progressing, life-threatening infection that primarily affects the soft tissues, particularly the fascial planes. While Group A *Streptococcus *is the most commonly identified pathogen, infections are often polymicrobial, involving organisms such as *Pseudomonas*, *Klebsiella*, and *Clostridium *species [4, 9]. These bacteria release enzymes and toxins that enable the infection to spread quickly, destroying tissue and compromising blood flow, which leads to ischemia and widespread necrosis [7, 8, 10]. NF tends to occur in individuals with weakened immune systems or those with chronic conditions, such as diabetes, obesity, or peripheral vascular disease. It often develops after skin trauma or surgical procedures, but can also arise through hematogenous spread [4]. Diagnosis can be challenging early on due to the poor vascular supply of the fascia, which may delay the appearance of noticeable signs. Timely surgical intervention is critical to limit progression and prevent severe complications such as sepsis or toxic shock syndrome.

Cellulitis is a superficial bacterial infection of the skin, typically arising from a breach in the skin barrier. It commonly presents with systemic symptoms, such as fever, chills, and fatigue, which can sometimes cause the patient to appear toxic [8, 9]. Patients may also exhibit altered mental status, tachycardia, and hypotension. The skin findings in cellulitis include widespread erythema with poorly defined borders, localized swelling, and often lymphadenitis [8, 9]. Mild blistering can occur but is uncommon. Pain and tenderness tend to be confined to the affected area [10]. The common clinical features of cellulitis are summarized in Table 1. When treated promptly with standard antibiotics, cellulitis generally resolves without severe complications, allowing patients to return to baseline health [10,11].

In contrast, NF is a rapidly advancing infection that affects deeper subcutaneous tissue and fascia. Early on, NF may mimic cellulitis but quickly progresses to more severe symptoms. Patients often present with fever, chills, fatigue, and systemic toxicity signs such as altered mental status, tachycardia, hypotension, and tachypnea [9]. Clinically, NF manifests with pronounced erythema, marked swelling, and necrotic skin patches with poorly defined margins. Vesicles or bullae with possible fluid discharge are common, while lymphadenitis is typically absent [9, 11]. Pain is often severe and disproportionately intense compared to physical findings, frequently extending beyond the visible skin involvement [9]. The clinical features of NF are summarized in Table 2. A hallmark sign of NF is crepitus, which is often caused by gas-producing bacteria, although it may not always be present [11]. Due to its aggressive nature, NF constitutes a medical emergency that requires immediate and aggressive intervention.

**Table 2:** Comparative Clinical Features of Cellulitis and Necrotizing Fasciitis Across Systemic, Cutaneous, and Pain-Related Domains This table compares the clinical features of cellulitis and necrotizing fasciitis, grouped by systemic, skin, and pain-related signs. Although both conditions may present with fever, fatigue, and erythema with poorly defined borders, necrotizing fasciitis is distinguished by findings such as tachypnea, necrotic skin changes, absence of lymphadenitis, and severe pain that exceeds physical exam findings. Recognizing these key differences is essential for prompt diagnosis and timely surgical intervention in necrotizing fasciitis [8-11].

Category	Clinical Feature	Cellulitis	Necrotizing Fasciitis
General	Fever/Chills	Present	Present
	Toxic appearance	Present	Present
	Fatigue	Present	Present
	Altered mental status	Possible	Possible
	Tachycardia	Present	Present
	Hypotension	Possible	Present
	Tachypnea	Not typical	Common
Skin	Erythema	Poorly demarcated borders	Poorly demarcated borders
	Edema	Tense edema	Tense edema with or without discharge
	Skin changes	Glossy	Necrotic changes possible
	Vesicles or bullae	Possible	Possible
	Lymphadenitis	Often present	Typically absent
	Necrosis	Not present	Necrosis common
Pain	Pain location	Localized to the infected area	Extends beyond the infected area
	Pain severity	Proportionate to the exam	Disproportionate to the exam findings

Recent advances have aimed to improve the timely diagnosis of NF through the development of objective, high-specificity models. The LRINEC score, originally developed using six laboratory parameters, remains a widely used tool. However, its sensitivity is variable and often inadequate in early presentations (43%-85%) despite a relatively high specificity (63%-93%) [12, 13]. To address this, modified versions such as the m-LRINEC and MLRINEC have demonstrated superior diagnostic accuracy. For instance, the MLRINEC score achieved a sensitivity of 91.8% and specificity of 88.4%, with an area under the curve (AUC) of 0.893, outperforming the classic model [14]. Similarly, the SIARI score integrates clinical features, such as pain severity and erythema, with laboratory data and has shown improved early detection of NF, yielding an AUC of 0.83 [15-17]. These models facilitate earlier diagnosis and help clinicians distinguish NF from less aggressive conditions such as cellulitis, particularly in cases with ambiguous presentations. Although clinical judgment remains paramount, the integration of these evidence-based tools into emergency protocols significantly enhances the early identification of NF and supports prompt surgical referral and intervention.

The LRINEC score was calculated for this patient and yielded a value of one, placing him in the low-risk category for NF. However, it is well recognized that the LRINEC score has important limitations, particularly its reduced sensitivity in early presentations or in patients with atypical clinical features. A low score does not reliably exclude the diagnosis, and reliance on this tool alone may delay appropriate treatment. In this case, despite the low LRINEC score, the patient’s rapid clinical deterioration and concerning local findings warranted a strong suspicion for NF. Given that clinical judgment remains the cornerstone of diagnosis and management, a decision was made to pursue urgent surgical debridement, which ultimately confirmed the diagnosis and facilitated timely intervention.

In this case report, the diagnosis of NF was established through a high degree of clinical suspicion. The initial evaluation included a CT scan, which demonstrated significant subcutaneous edema and confirmed an intense inflammatory process in the left lower limb [8, 9, 11]. Imaging studies such as CT play a crucial role in delineating the extent of infection and guiding management decisions. The prognosis for patients with NF largely depends on the timeliness of diagnosis and the prompt initiation of comprehensive, multidisciplinary treatment. Effective management involves broad-spectrum intravenous antibiotics targeting the causative pathogens, combined with urgent surgical debridement to remove necrotic tissue [10, 12]. Ongoing wound assessment, including meticulous irrigation and repeated aggressive debridement as necessary, is crucial for controlling infection and promoting healing. This integrated approach has significantly decreased mortality rates in affected patients [11, 12]. Consequently, rapid diagnosis, decisive surgical intervention, and comprehensive supportive care remain critical to improving outcomes in this life-threatening condition.

## Conclusions

This case underscores the importance of early recognition and timely intervention in NF, particularly when presentations initially mimic cellulitis or other benign soft tissue infections. Despite a low LRINEC score, the patient’s rapid clinical decline and evolving cutaneous findings necessitated urgent surgical exploration, which ultimately confirmed the diagnosis. This outcome highlights the limitations of predictive models in early disease and reinforces that clinical judgment remains the most reliable determinant in guiding management. Aggressive surgical debridement, appropriate antimicrobial therapy, and coordinated multidisciplinary care, including wound reconstruction and rehabilitation, were pivotal in achieving a favorable outcome. Clinicians must maintain a high index of suspicion in patients with atypical or rapidly progressive infections, as timely operative management remains the cornerstone of reducing morbidity and mortality in NF.
